# Rat pulmonary responses to inhaled nano-TiO_2_: effect of primary particle size and agglomeration state

**DOI:** 10.1186/1743-8977-10-48

**Published:** 2013-10-04

**Authors:** Alexandra Noël, Michel Charbonneau, Yves Cloutier, Robert Tardif, Ginette Truchon

**Affiliations:** 1 Département de santé environnementale et de santé au travail, Institut de recherche en santé publique, Université de Montréal, C.P. 6128 Succursale Centre-Ville, Montréal, Québec H3C 3J7, Canada; 2 INRS-Institut Armand-Frappier, Université du Québec, 531 Boul. des Prairies, Laval, Québec H7V 1B7, Canada; 3 Institut de recherche Robert-Sauvé en santé et en sécurité du travail (IRSST), 505 Boul. De Maisonneuve Ouest, Montréal, Québec H3A 3C2, Canada

**Keywords:** TiO_2_ nanoparticles, Inhalation, Agglomeration state, Primary particle size, Inflammation, Cytotoxicity, Oxidative stress

## Abstract

**Background:**

The exact role of primary nanoparticle (NP) size and their degree of agglomeration in aerosols on the determination of pulmonary effects is still poorly understood. Smaller NP are thought to have greater biological reactivity, but their level of agglomeration in an aerosol may also have an impact on pulmonary response. The aim of this study was to investigate the role of primary NP size and the agglomeration state in aerosols, using well-characterized TiO_2_ NP, on their relative pulmonary toxicity, through inflammatory, cytotoxic and oxidative stress effects in Fisher 344 male rats.

**Methods:**

Three different sizes of TiO_2_ NP, i.e., 5, 10–30 or 50 nm, were inhaled as small (SA) (< 100 nm) or large agglomerates (LA) (> 100 nm) at 20 mg/m^3^ for 6 hours.

**Results:**

Compared to the controls, bronchoalveolar lavage fluids (BALF) showed that LA aerosols induced an acute inflammatory response, characterized by a significant increase in the number of neutrophils, while SA aerosols produced significant oxidative stress damages and cytotoxicity. Data also demonstrate that for an agglomeration state smaller than 100 nm, the 5 nm particles caused a significant increase in cytotoxic effects compared to controls (assessed by an increase in LDH activity), while oxidative damage measured by 8-isoprostane concentration was less when compared to 10–30 and 50 nm particles. In both SA and LA aerosols, the 10–30 nm TiO_2_ NP size induced the most pronounced pro-inflammatory effects compared to controls.

**Conclusions:**

Overall, this study showed that initial NP size and agglomeration state are key determinants of nano-TiO_2_ lung inflammatory reaction, cytotoxic and oxidative stress induced effects.

## Background

In recent decades, nanoparticles (NP) (< 100 nm) have attracted the increased attention of the industrial sector due to their unique physico-chemical properties and numerous applications. Indeed, compared to their larger-sized counterparts, NP have improved and distinctive surface characteristics which are considered as the building blocks of nanotechnology. This fast developing field is expected to generate by 2014 more than 10 million jobs related to this technology [1]. Thus, an increasing number of workers are going to be handling NP during the production and disposal of several consumer products [2]. Reliable testing strategies to investigate possible health effects caused by NP exposure are therefore urgently needed. Risk assessment is based on exposure expressed in terms of dosimetry and toxicological data [3]. Hence, research studies should examine the characteristics of NP or aerosols that determine their ability to cause deleterious effects. Currently, the best metric, such as mass, surface area, number or size distribution, to measure NP in order to evaluate risk and prevent the development of occupational diseases is still a matter of debate [3].

Several toxicological studies have addressed the micro versus the nano size effect. This has indicated at equivalent mass concentration that agglomerated NP produce greater pulmonary inflammation responses than micron size particles [4-8]. However, few studies have investigated *in vivo* the size-dependent effects of NP [9-11]. As NP (< 100 nm) size decreases, it is expected that the percentage of atoms and active sites at the surface, as well as the structural imperfections, increase significantly [12,13]. In addition, it has previously been shown that metal oxide particles less than 30 nm in size, had enhanced interfacial reactivity [14,15]. Correspondingly, the biological reactivity of smaller NP is expected to be higher than that of larger NP [16]. Moreover, it is at approximately 20 nm that the highest relative deposition efficiency of NP in the alveolar region occurs [17,18]. This suggests that NP of different initial sizes could produce different biological responses [16,19].

TiO_2_ is a substance that is manufactured at a large scale, either as crude, fine or ultrafine powder. Given its important industrial production, several countries use TiO_2_ as a reference nanomaterial in research and in the assessment of workplace exposures [20-23]. However, to this day there is still limited consistent toxicological data and similarly limited epidemiological studies related to occupational exposures to this chemical [24-29]. This puts the emphasis on the need for more research on TiO_2_ NP.

Our research group and other studies have previously reported that agglomeration is a process occurring in nano-TiO_2_ aerosol production [9,19,30-34]. Using different methods to generate NP aerosols in laboratory studies, we have shown that a given TiO_2_ NP can agglomerate in different sizes and structures [33]. This observation is relevant and valuable for toxicological assessment since there is increasing evidence that aerosol characteristics can modulate NP interaction with biological systems [9,18,35-38]. Indeed, agglomeration of NP has multiple influences on dose characteristics and on their pulmonary toxicity and kinetics. It can affect: 1- the number concentration in the aerosol; 2- the deposition site in the respiratory tract; 3- the possibility of detection and subsequent phagocytosis by alveolar macrophages; 4- the fate in lung tissue and translocation to other extra-pulmonary compartments; and 5- the size, density, shape and structure of the resulting particle, being either loose or compact. Furthermore, once deposited in the respiratory tract, NP can interact with biological material (e.g., pulmonary surfactant, macromolecules and proteins), which can also have an impact by increasing, slowing or preventing the degree of agglomeration in the physiological environment [36,39,40]. Taken together, it is highly relevant to characterize and consider the agglomeration state of NP, particularly metal oxides, in nanotoxicological studies.

We have previously reported that rats exposed by inhalation to small agglomerates (< 100 nm) of 5 nm TiO_2_ showed greater cytotoxic and oxidative stress responses than rats exposed to larger agglomerates (> 100 nm) of the same NP, which induced a slight inflammatory reaction [41]. This suggested that biological responses to TiO_2_ might depend on the dimension and concentration of the NP agglomerates. These results are in line with the current hypothetical mechanisms of NP pulmonary toxicity, based on the inflammation and oxidative stress paradigm [17,42-44].

In inhalation studies, establishing the effect of primary NP size involves considering the size of the agglomerates, and only few studies have addressed this issue. In the early 90’s, several influential studies [4,5,45,46] were conducted and showed differences in toxicologically relevant responses and translocation of NP versus submicrometer particles administered as submicrometer-sized aerosols. A recent study conducted by Balasubramanian *et al.*[47] showed in rats that primary NP size of inhaled gold NP influenced the whole-body biodistribution. Gold NP of 7 or 20 nm primary sizes were generated into aerosols of similar number concentration and size distribution. This showed that the agglomerates of the 7 nm NP were distributed in more organs than the 20 nm NP and that macrophage clearance was more effective for agglomerates of larger primary size. In a study by Pauluhn [11], rats exposed to aluminum oxide NP aerosols showed a clearance half-time which increase with decreased primary NP size (10 nm - 840 nm in aerosols and 40 nm – 660 nm in aerosols), while the inflammatory response appeared to be determined by the size of the agglomerates. In a study conducted by Grassian *et al.*[9], mice exposed to 7 mg/m^3^ of 5 nm TiO_2_ NP (120 nm in aerosol) or 21 nm NP (139 nm in aerosol) showed that the latter was slightly, but significantly more toxic than the smaller NP. These authors suggested that the difference in the structure of the agglomerates, which was compact for the 5 nm TiO_2_ versus loose for the 21 nm particles, could be partly responsible for the results. Collectively, these inhalation nanotoxicology studies suggest that smaller initial NP size could distribute in more extra-pulmonary organs and might not always induce enhanced lung inflammatory reactions when compared to larger nanometric counterparts.

The exact role of primary NP size and their degree of agglomeration in aerosols in the determination of pulmonary effects is still poorly understood. Indeed, smaller NP are thought to have greater biological reactivity, but the level to which these NP agglomerate in aerosols may also have an impact on the pulmonary response. Moreover, NP of different primary sizes could possibly bundle in agglomerates of various dimensions, shapes and structures in aerosols, which can also influence their pulmonary toxicity and kinetics. Thus, the primary particle size and agglomeration state of NP can be of significance in toxicity and partly responsible for the distinct effects induced in lungs. To our knowledge, no previous study has addressed both factors simultaneously, i.e., the size-dependent effect of NP while also comparing the influence of the agglomeration state. Elucidating the impact of these factors on the mechanism of toxicity will provide important knowledge for NP risk assessment.

The aim of this study was to investigate the role of primary NP size and agglomeration state in aerosols using well-characterized TiO_2_ NP and to compare their relative pulmonary toxicity, through inflammatory, cytotoxic and oxidative stress effects in rats. For this purpose, we used three different sizes of TiO_2_ NP, i.e., 5, 10–30 or 50 nm inhaled as small (< 100 nm) or large agglomerates (> 100 nm) at a concentration of 20 mg/m^3^.

## Results

### Characterization of initial NP powders

In this paper, we define nanoparticle (NP) as particle whose nominal diameter (geometric, mobility, aerodynamic, projected surface or other) is less than 100 nm [48]. The characterization of the three nano-TiO_2_ powders (as received by the manufacturers) which were used to produce the aerosols along with transmission electron microscopy (TEM) images, are presented in Table 1. X-ray spectrometric analysis (EDS) analysis showed that all powders were made of TiO_2_. The size distribution of the NP present in the powders was established by measuring the diameter of over 300 particles by TEM. These results showed that the median diameter of the NP present in the powder labeled by the manufacturer as 5 nm was 10.6 nm, while it was 18.2 nm and 34.8 nm for the 10–30 nm and 50 nm powders, respectively. The analysis also showed that the size distributions of the three nano-powders slightly overlapped (data not shown). In addition, TEM analysis showed that, in the case of the 5 and 50 nm powders, particles had a spherical or rod-shaped morphology, while the 10–30 nm powder, showed only spherical particles. Analysis by X-ray diffraction (XRD) revealed that the 5 and 10–30 nm powders were predominantly in the anatase form (> 97%), while the 50 nm powder showed a slightly higher presence of the rutile form (< 20%).

**Table 1:** **TiO**_**2 **_**NP powder characterization assessed by transmission electron microscopy (TEM) and X-ray diffraction (XRD)**

	**Parameters**	**Methods**	**Results**
5 nm	Morphology	TEM	Spherical: > 97%
n = 375			Rod-shape: < 3%
			
	Crystal phase	XRD	Anatase: > 97% (volume)
			Rutile: < 3%
	Size (nm)	TEM	53.3% between 3 and 11
			Median: 10.6
			Min: 3.9 and max: 75.6
			
10-30 nm	Morphology	TEM	Spherical
n = 330	Crystal phase	XRD	Anatase: 100% (volume)
			No rutile detected
			
	Size (nm)	TEM	67.6% between 11 and 31
			Median: 18.2
			Min: 4.9 and max: 70
50 nm	Morphology	TEM	Spherical: > 80%
n = 302			Rod-shape: < 20%
	Crystal phase	XRD	Anatase: > 80% (volume)
	Size (nm)	TEM	Rutile: < 20%
			47% between 31 and 61
			Median: 34.8
			Min: 8.6 and max: 112

**Table 2:** Measurements and characterization of the NP aerosols

**Experimental groups**							
**Parameters**	**Control**	**5 nm SA**^**a**^	**5 nm LA**^**b**^	**10-30 nm SA**	**10-30 nm LA**	**50 nm SA**	**50 nm LA**
Average mass concentration^c^ (mg/m^3^)	0.05	18.77	19.30	22.04	21.99	21.38	21.94
Min and max^d^ (mg/m^3^)	0.03 and 0.27	17.12 and 22.47	17.43 and 22.18	18.83 and 27.99	20.48 and 25.42	19.66 and 25.34	20.45 and 30.09
Total particle number^e^ (/cm^3^)	-	3 159 758	308 098	1 808 939	374 225	1 320 239	280 379
D_25_^e,f^ (nm)	-	29	156	28	128	35	135
NMAD^*^ or D_50_^e,f^ (nm)	-	48	369	65	255	85	321
GSD^g^	-	3.1	2.2	3.3	2.6	3.2	2.6
D_75_^e,f^ (nm)	-	124	575	183	686	305	783
Fraction of NP agglomerates <100 nm^e^ (%)	-	71	16	63	18	54	19

^a^ Aerosol composed of small agglomerates.

^b^ Aerosol composed of large agglomerates.

^c^ Average mass concentration determined by weight measurements.

^d^ Min and max concentration determined by a DustTrak.

^e^ Measurements made with the ELPI.

^f^ Aerodynamic diameters for which 25% (D_25_), 50% (D_50_ or NMAD) or 75% (D_75_) of the particles in the aerosol are smaller than this value, based on number concentration. D_75_ – D_25_ is the interquartile range and represents the size distribution where we find 50% of the particles.

^g^ GSD: geometric standard deviation.

^*^NMAD: number median aerodynamic diameter.

### Measurement and characterization of NP aerosols

**Figure 1:**
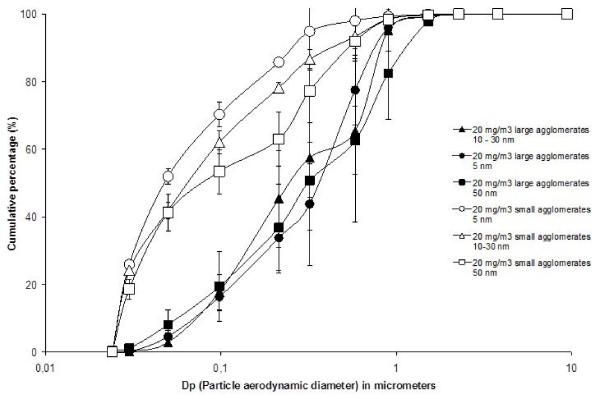
**Cumulative distributions based on number concentration of the NP aerosols.** NP aerosols cumulative size distributions based on number concentration measured with the ELPI. For each aerosol 5 thirty-minute samples were collected every hour of the experiment.

Throughout this article, we refer to small agglomerate (SA) aerosols as mainly composed of agglomerates with a size smaller than 100 nm and large agglomerate (LA) aerosols as essentially composed of agglomerates larger than 100 nm. Aerosol’s characterization data carried out with the ELPI, DustTrak and gravimetric measurements are presented in Table 2. Cumulative size distributions based on number concentration measured with the ELPI are shown in Figure 1. Three values were used to estimate the number size distributions, namely the first quartile (D_25_), the midpoint (D_50_) or the median aerodynamic diameter based on the number concentration (NMAD), and the third quartile (D_75_). These results show that for each TiO_2_ NP, the size distributions obtained for SA and LA aerosols are different (Table 2 and Figure 1). The targeted exposure concentration was 20 mg/m^3^ for all exposed groups and the concentrations gravimetrically measured were between 18.8 and 22.0 mg/m^3^. As measured with the ELPI, the total particle number was higher in the aerosols composed of SA compared to the aerosols composed of LA.

**Figure 2:**
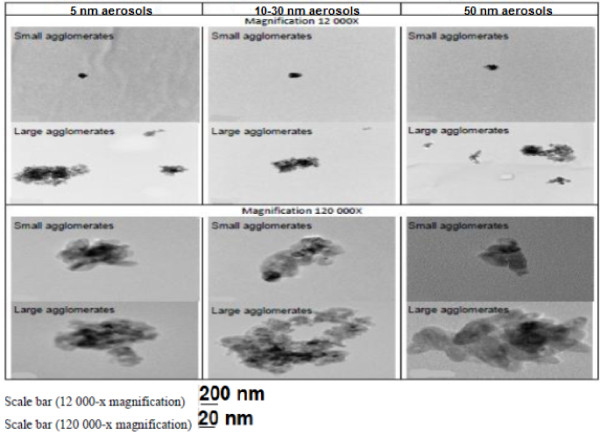
**NP aerosol agglomerate structure observed by transmission electron microscopy.** Air samples were collected on pre-carbon coated Formvar copper grids glued onto 25-mm polycarbonate filters. Characterization (shape, agglomeration degree and structure) of the nano-aerosols was performed by TEM.

Figure 2 shows representative TEM images of particles from the aerosols composed of SA or LA. For the same initial particle size, TEM images (12 000-×) allowed the observation of qualitative differences in the size, shape and structure of the agglomerates present in the aerosols (Figure 2). Indeed, SA aerosols were mostly composed of small compact agglomerates, while LA aerosols had larger agglomerates with void space. Higher magnification (120 000-×) allowed the observation of irregularly shaped small agglomerates (Figure 2).

### Pulmonary deposition

**Figure 3:**
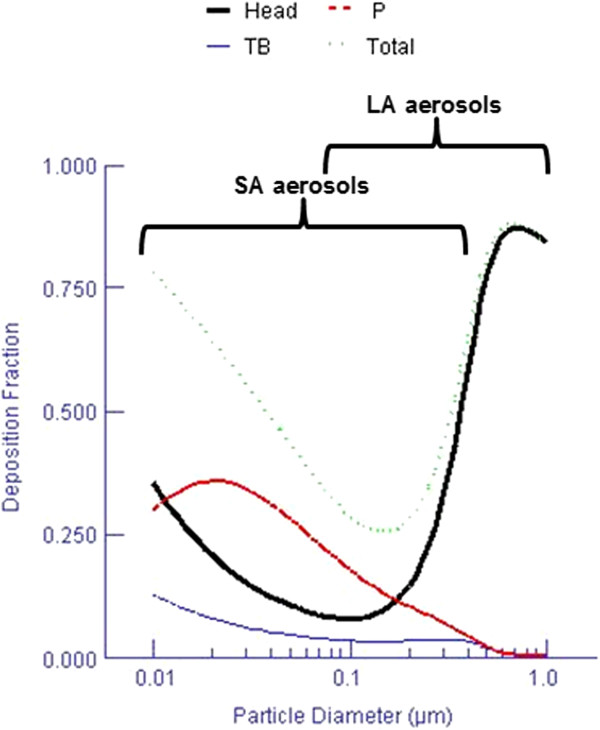
**Computed fractional deposition of NP agglomerates in the rat respiratory tract.** Estimated deposition fraction for the aerosols at 20 mg/m^3^ composed of SA or LA modeled for multiple diameters. TB = tracheobronchial; P = pulmonary. Functional residual capacity (FRC) volume 4 ml; head volume: 0.42 ml; nasal breathing route; tidal volume 2.1 ml; breathing frequency 110/min and inspiratory fraction 0.1.

Using the polydisperse diameter interquartile ranges presented in Table 2, the estimated respiratory tract deposition fraction of NP agglomerates was computed with the MPPD model for LA and SA aerosols. These results are presented in Figure 3 and indicate that pulmonary deposition is different for the two types of aerosols.

**Table 3:** BALF cytology and estimation of NP endocytosis by macrophages following inhalation exposure

***Experimental groups***							
**Cell type**	**Control**	**5 nm SA**	**5 nm LA**	**10-30 nm SA**	**10-30 nm LA**	**50 nm SA**	**50 nm LA**
**Total cells**^**a**^	2.36±0.46	4.32±0.27*	4.02±0.63	4.41±0.40*	5.38±0.45**	3.01±0.36	4.82±0.36**
**Lymphocytes**^**b**^	4.16±0.60	2.15±0.37	8.08±1.97	4.57±0.82	7.02±2.67	2.85±0.49	4.34±1.37
**Neutrophils**^**c**^	0.23±0.05	1.18±0.19	1.18±0.34	1.26±0.16	1.47±0.41*	0.84±0.18	1.78±0.22**
**Macrophages**^**a**^	2.30±0.45	4.18±0.27*	3.82±0.59	4.24±0.39*	5.16±0.42**	2.90±0.35	4.60±0.36**
**Estimation of endocytosis (%)**^**d**^	---	53±3.8^ŧ^	78±1.3	53±1.9^ŧ^	78±2.9	65±1.9^ŧ^	83±1.3

^a^ number of cells x 10^6^ ± standard error on the mean (SEM); ^b^ number of cells x 10^4^ ± SEM; ^c^ number of cells x 10^5^ ± SEM; ^d^ estimation of the extend of NP phagocytosis by macrophages. Mean values (n = 6 rats per group) significantly different from controls (ANOVA followed by a Tukey’s test) *p<0.05, **p<0.01, ^ŧ^p<0.05 significantly different from LA aerosol of the same primary NP size.

### Rat pulmonary response

**Figure 4:**
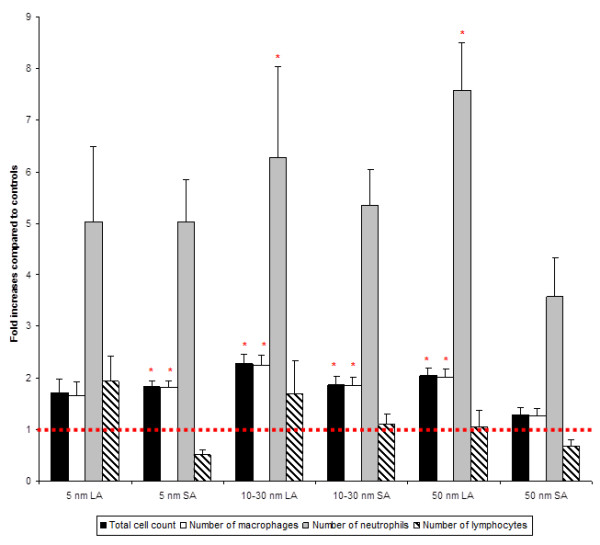
**BALF cytology analyzed by the cytospin method.** Data were expressed as fold increases of exposed groups compared to controls. Bars represent the mean value and the standard error of the mean obtained for 6 rats in each exposure group. Statistical procedures: ANOVA followed by a Tukey’s test. *Mean value is statistically different from control level p < 0.05.

A transient increase in leukocytes from BALF is a natural lung defense mechanism following deposition of inhaled particles. In this manner, BALF cytology analysis from rats exposed to SA aerosols showed increases in total cell count, number of macrophages and neutrophils compared to the control group. Increases were slightly but statistically significant (p < 0.05) for total cell count and number of macrophages in the 5 and 10–30 nm groups compared to the controls (Table 3, Figure 4). Rats exposed to LA aerosols also showed increases in total cell count, number of macrophages and neutrophils compared to the control group. Results were significant (p < 0.05) for total cell count, number of macrophages and neutrophils in the 10–30 and 50 nm groups compared to the controls (Table 3, Figure 4).

**Figure 5:**
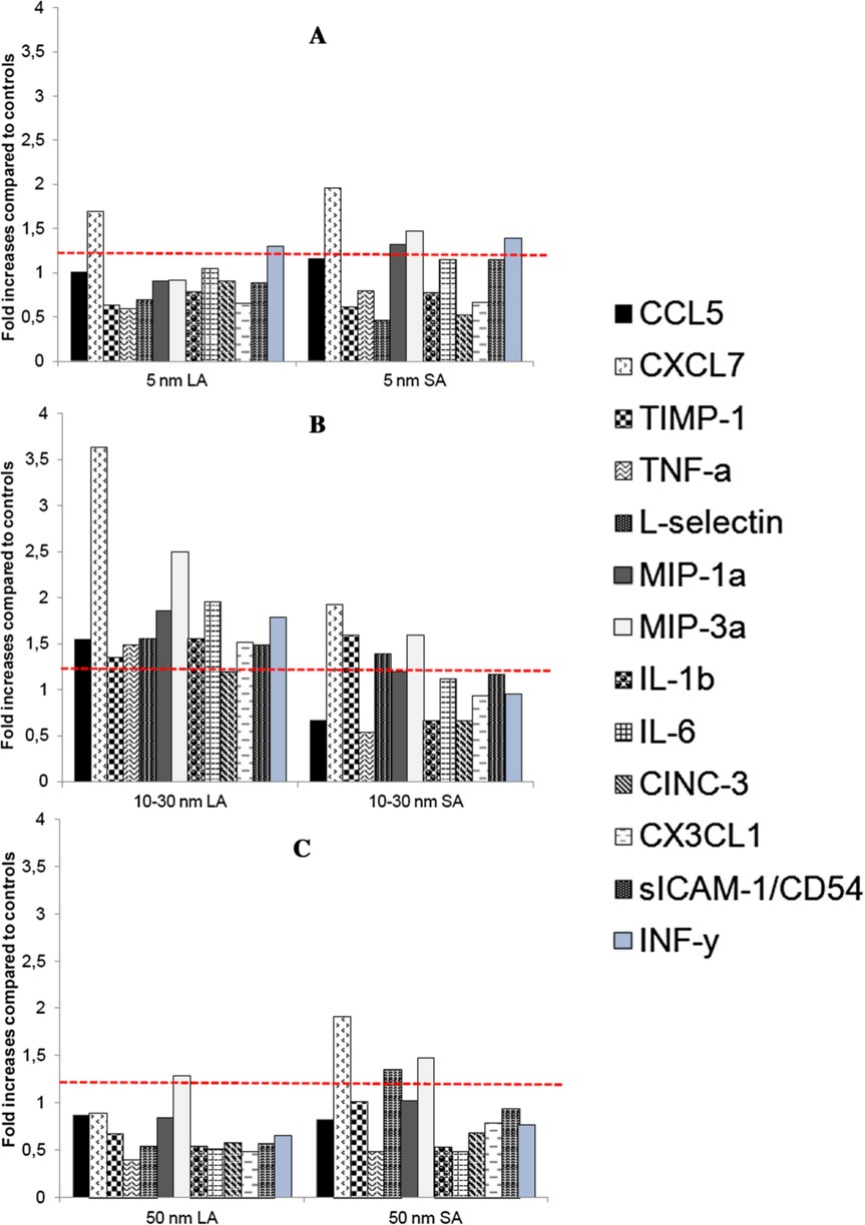
**Relative levels of BALF pro-inflammatory cytokines.** BALF pro-inflammatory cytokines were expressed as fold increases of exposed groups compared to controls. Samples from all rats of the same exposure group were pooled together. The ratio for each cytokine was calculated as described in materials and methods. Results with ratios ≥1.2 were considered to represent a slight inflammation. **(A)** Aerosols with a primary NP size of 5 nm. **(B)** Aerosols with a primary NP size of 10–30 nm. **(C)** Aerosols with a primary particle size of 50 nm.

The profiles of inflammatory cytokines are shown in Figure 5. Thirteen of the 29 cytokines analyzed in the assay showed ≥ 1.2-fold increases compared to the control group (CCL5, CXCL7, TIMP-1, TNF-α, L-selectin, MIP-1α, MIP-3α, IL-1β, IL-6, CINC-3, CXCL1, sICAM, INF-γ). Noticeably, the 10–30 nm LA aerosol showed a profile where all of the 13 cytokines were increased compared to the controls.

**Figure 6:**
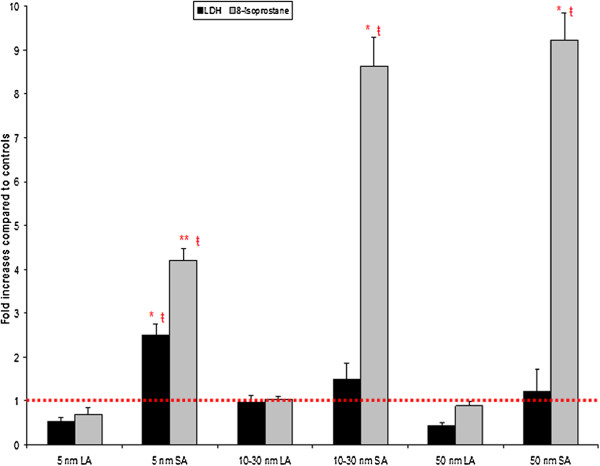
**BALF cytotoxicity (LDH) and oxidative stress (8-isoprostane) markers.** Data were expressed as fold increases of exposed groups compared to controls. Bars represent the mean value and the standard error on the mean obtained for 6 rats in each exposure group. LDH assay for each rat was done in triplicate and 8-isoprostane was in duplicate. Statistical procedures: ANOVA followed by a Tukey’s test. * Mean value is statistically different from control level p < 0.05; ** Mean value is statistically different from control level and 10–30; 50 nm SA aerosols p < 0.05; ŧ Mean value is statistically different from all LA aerosol levels p < 0.05.

8-isoprostane, a marker of oxidative stress, showed a statistically significant increase for all SA aerosols compared to the controls or respective LA aerosols (Figure 6). There were also significant differences between the three SA aerosols. For the 5 nm SA aerosol the 8-isoprostane concentration was significantly (p < 0.05) lower compared to the 10–30 and 50 nm SA aerosols (Figure 6). For LDH activity, only the results for the 5 nm SA aerosol were statistically (p < 0.05) different from the controls (Figure 6). There was also a significant (p < 0.05) difference between the 5 nm SA and all LA aerosols (Figure 6).

**Figure 7:**
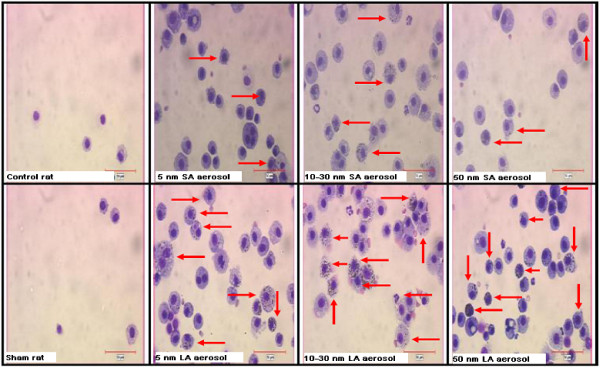
**Representative cell morphology from BALF cytopreparations of rats.** Optical microscopy (magnification 400-x) of cells cytopreparation collected in BALF of sham, controls (exposed to compressed air) and TiO_2_ NP exposed rats. For all TiO_2_ exposed groups, the images show giant foamy macrophages and the distribution of macrophages containing phagocytized TiO_2_ NP (arrows). Scale bar = 50 μm.

Representative cell morphology of the BALF cytopreparations from the control and exposed rats are shown in Figure 7. The control group showed typical BAL cells. For all nano-TiO_2_ exposed groups, the majority of the cells (macrophages, neutrophils and lymphocytes) were also typical, although giant cells (macrophages) and fragmented nucleus macrophages were observed (Figure 7).

### Estimation of NP endocytosis by macrophages

Figure 7 shows representative images of BAL macrophages with (arrows) or without phagocytized NP agglomerates. All groups exposed to the LA aerosols had 79 ± 2% (standard error on the mean, SEM) of macrophages that contained nano-TiO_2_ agglomerates, whereas for the SA aerosols, it was 57 ± 3% (SEM) (Table 3). For each primary NP size, a significant difference was observed for the percentage of particle-laden macrophages between the LA and SA aerosols (Table 3).

### Lung histopathology

**Figure 8:**
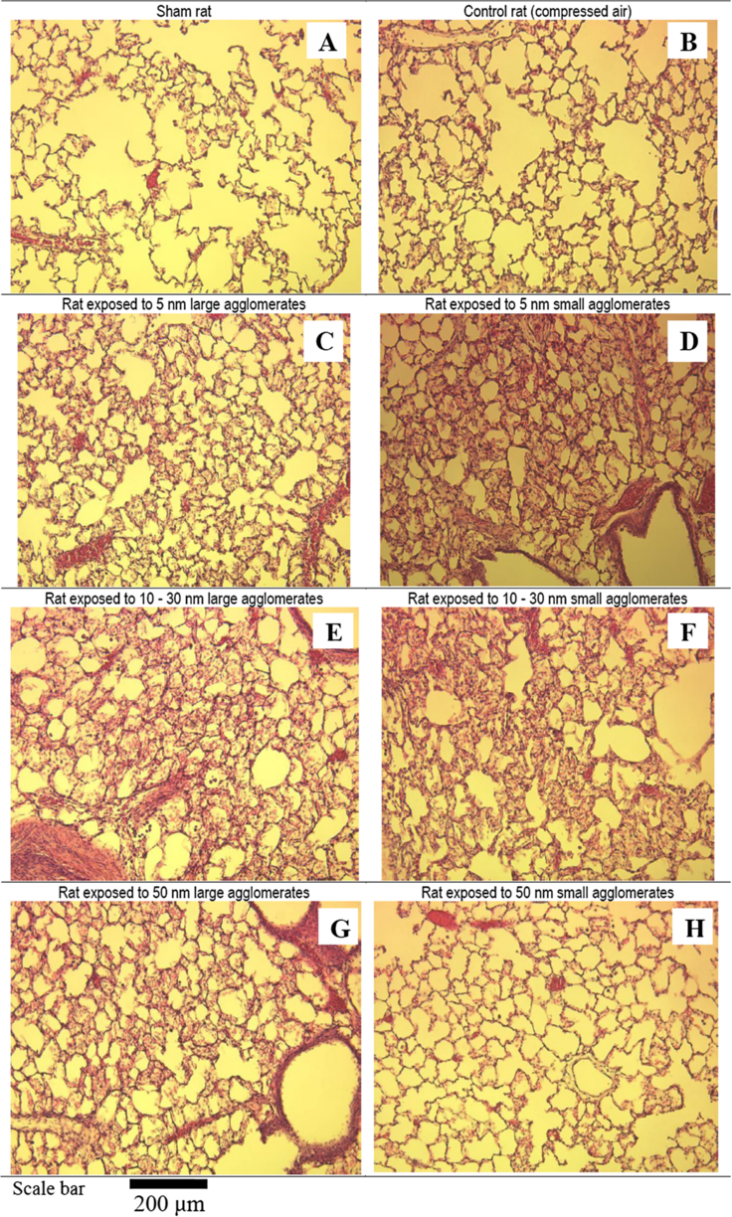
**Optical microscopy images (100-x) of lung tissue sections.** Morphological assessments of lung tissue stained with haematoxylin and eosin of sham **(A)**, control (exposed to compressed air) and TiO_2_ NP exposed rats by means of inhalation for 6 hours. Responses to nano-TiO_2_**(C to G)** were different in intensity compared to the controls **(B)**, except for the 50 nm SA group **(H)**. The lungs of rats exposed to 5 and 10–30 nm SA aerosols **(D and F)** as well as rats exposed to 10–30 and 50 nm LA aerosols **(E and G)** showed more leukocyte infiltration compared to the control group **(B)**.

**Figure 9:**
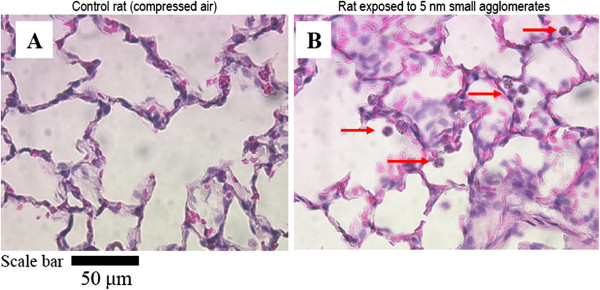
**Optical microscopy images (400-x) of lung tissue sections. (A)** Lung of control rat exposed to compressed air. **(B)** Lung of a rat exposed to small agglomerates of 5 nm NP. This figure demonstrates TiO_2_ NP engulfed by alveolar macrophages (arrows).

Figures 8 and 9 show images of haematoxylin and eosin stained lungs. The control group did not show any signs of inflammation. Noteworthy, the cellular influx observed were partial since these lungs were also used for BAL. Notwithstanding, morphological assessments of lung tissue responses to nano-TiO_2_ were different in intensity compared to the controls, except for the 50 nm SA group (Figure 8). The lungs of rats exposed to 5 and 10–30 nm SA aerosols as well as rats exposed to 10–30 and 50 nm LA aerosols showed more leukocyte infiltration compared to the control group (Figure 8). This is consistent with the cytological analysis (Table 3, Figure 4) and is considered to represent a normal macrophage clearance response. Figure 9, which is representative of all groups, shows TiO_2_ NP-laden macrophages in the 5 nm SA aerosol group.

## Discussion

Since TiO_2_ NP tends to agglomerate in aerosols [9,32-34], establishing the effect of these NP involves considering the primary particle size and that of the agglomerates [9]. The agglomeration state of NP influences the site of particle deposition in the respiratory tract and affects lung clearance mechanisms, including endocytosis [9,19,45-53]. In general, the size distribution of a NP aerosol is composed of a less (< 100 nm) and highly (> 100 nm) agglomerated fraction, with the percentage varying from one aerosol to another. The less agglomerated fraction, for which the size of the agglomerates is closer to the size of the primary NP, could possibly induce effects related to their interaction with lung tissue and epithelial cells at the site of pulmonary deposition. These small particles are also more readily available for translocation to the lymph nodes or bloodstream [17]. Larger agglomerates (> 100 nm) are thought to be more easily detected and removed by the lung macrophages [41,54-57].

In our study, the cellular pulmonary response observed for all exposed groups denoted by the increases in leukocytes from BALF compared to the controls (Table 3, Figure 4) could be considered as a normal immediate response to particle aggression [58]. The increase in the number of macrophages and neutrophils is thought to contribute to particle removal. Indeed, previous studies have shown that following inhalation of nano-TiO_2_ this type of response is temporary and resolves rapidly [9,19,30,32]. Hence, after an acute exposure, this could be a defense mechanism [59].

### NP aerosol characterization

For the LA aerosols, we observed that the 5 nm TiO_2_ produced larger agglomerates than the 10–30 and 50 nm particles (Table 2). This result could be partly explained by the fact that as particle size decreases, the attractive force per unit mass increases, which favors agglomeration [60]. Indeed, small particles that coagulate into agglomerates larger than superior-sized counterparts is a common finding previously described in numerous inhalation studies, including ones using nano-TiO_2_[11,30,38,45,61].

### LA aerosols

As shown in Figure 3, the estimated pulmonary deposition was different for the LA and SA aerosols. Also, given their different agglomeration states (Table 2), it can be assumed, as described by Oberdörster *et al.*[17] as well as Geiser and Kreyling [62], that the penetration of these aerosols into the various regions of the respiratory tract is different. Considering their size distribution characteristics (D_25_ = 128 nm to D_75_ = 783 nm; Table 2), particles of the LA aerosols could be more easily detected by immune system cells, including alveolar macrophages (Table 3, Figures 7 and 8). Indeed, the estimation of NP endocytosis showed for all LA aerosols that 79 ± 2% of macrophages contains nano-TiO_2_ agglomerates (Table 3). In our study, activation of macrophages following phagocytosis of large NP agglomerates (Figure 7) is supported by a slight but significant increase in the total cell count, and number of macrophages and neutrophils compared to controls for two out of the three LA aerosols (10–30 and 50 nm) (Table 3 and Figure 4). However, the 50 nm LA aerosol did not increase the level of inflammatory cytokines, whereas interferon γ (INF-γ), chemokine C-X-C motif ligand 7 (CXCL7), interleukine-6 (IL-6), macrophage inflammatory protein 1α (MIP-1α) and MIP-3α were increased for the 10–30 nm LA aerosol when compared to the controls. These cytokines are produced by activated macrophages and act in host defense by promoting phagocytosis, resulting in chemotaxis, inflammatory cell recruitment and activation at the site of injury [63-65]. In particular, MIP-1α activates granulocytes (neutrophils, eosinophils, basophils) which can lead to acute neutrophilic inflammation [63-65]. The detection and phagocytosis of these agglomerates by macrophages can prevent their interaction with lung cells and tissue [41,45,66,67]. This is also supported by our results, where no cytotoxicity or oxidative stress effects, evaluated through LDH activity and 8-isoprostane concentration, were observed for these LA aerosols (Figure 6). LDH is a cytoplasmic enzyme that is released by dead cells and is therefore a suitable marker of cell cytotoxicity, while 8-isoprostane is a biomarker of lipid peroxidation and thus an indicator of oxidative stress effects [68,69].

An increase in the number of neutrophils supports the presence of an inflammatory reaction [12,32,39,65]. Thus, a mild significant inflammatory (p < 0.05) response was observed following the 10–30 and 50 nm LA aerosols exposures. These results are consistent with the common finding of various nanotoxicological studies on the increases in the number of neutrophils following agglomerated nano-TiO_2_ exposures [9,19,32,41,45,70-72]. Previous studies have also observed that inhalation of agglomerated nano-TiO_2_, in mice and rats, caused slight inflammatory responses and long-term pulmonary inflammation [9,19,30,32,72-75].

### SA aerosols

For the 5 and 10–30 nm SA aerosols, the cytological analysis showed a statistically significant increase in total cell count and number of macrophages (Table 3, Figure 4). Qualitatively, these results are consistent with the histopathological findings (Figure 8). The estimation of NP endocytosis showed that all SA aerosols had 57 ± 3% of macrophages containing nano-TiO_2_ agglomerates (Table 3). Despite the NMAD values that were below 100 nm for these aerosols, the D_75_ values ranged from 124 to 305 nm. Thus, the agglomerated (> 100 nm) fraction, which was encountered for 29 to 46% of these aerosols could explain the NP endocytosis observed. Nonetheless, for each primary NP size, a significant difference was observed for the percentage of particle-laden macrophages between LA and SA aerosols. Also, increases in the relative levels of CXCL7 and MIP-3α were observed in all SA aerosols, while it was also the case for the tissue inhibitor matrix proteinase 1 (TIMP-1), a glycoprotein involved in the degradation of the extracellular matrix, for the 10–30 nm SA aerosol. Considering the size distributions of the SA aerosols (D_25_ = 28 nm to D_75_ = 305 nm; Table 2) and as shown with the estimation of NP endocytosis, it can be assumed that these aerosols were not as well detected and phagocytized by alveolar macrophages as the LA aerosols. Thus, increased NP interaction with biological materials (lung cells and tissue) may have occurred compared to the LA aerosols and could be expressed as cytotoxicity and oxidative stress effects. In our study, statistically significant increases were observed in LDH activity and 8-isoprostane concentration for the 5 nm SA aerosol compared to the controls and its respective LA aerosol, while only 8-isoprostane was significantly increased for the 10–30 and 50 nm SA aerosols (Figure 6). Therefore, overall, the results for the SA aerosols indicate clear trends of NP interaction with lung cells and tissue through oxidative stress damage and suggestive slight cytotoxic effects (Figure 6).

### Effect of the agglomeration state

Overall, these results confirm, at a higher mass concentration, what we had previously shown at 7 mg/m^3^[41], namely that an acute inhalation of nano-TiO_2_ with two distinct agglomeration states, smaller or larger than 100 nm, induced different mild pulmonary effects. An acute inflammatory response measured by an increase in the number of neutrophils was induced by exposure to two out of three LA (> 100 nm) aerosols, while significant oxidative stress effects were observed after exposures to all of the SA (< 100 nm) aerosols. With respect to hazard identification, our results indicate that even though LA aerosols induced an acute inflammatory response, which is reversible according to the literature [9,19,32,45,70,72], it cannot be concluded that these aerosols induce toxicity through the same mechanisms as SA aerosols, which showed clear oxidative stress damage in BALF.

### Effect of primary nanoparticle size

For the three initial TiO_2_ NP sizes, we observed only one significant difference within the smaller than 100 nm agglomeration state aerosols. The significant difference was observed between the 5 nm and the two other SA aerosols for the 8-isoprostane concentration (Figure 6). This suggests that the larger 10–30 and 50 nm particles induced more lipid peroxidation and oxidative stress damage than the smaller 5 nm particles. Numerous inhalation studies have previously demonstrated that translocation of various type of NP, including TiO_2_, to extrapulmonary compartments occurred for small agglomerated NP (average diameter < 80 nm) in aerosols [37,76-83]. Collectively, these studies indicate that the penetration efficiency of NP through cellular membranes increases as the NP size decreases and that the translocation time increases with particle size [84-86]. Thus, in our study, the smaller size of the 5 nm particles (D_50_ = 48 nm in aerosol) would facilitate their possible and rapid translocation from the lung epithelial cells, thereby reducing their availability and time to cause cellular membrane lipid peroxidation at the NP - cell interface. The larger size of the 10–30 and 50 nm particles (D_50_ = 65 and 85 nm in aerosols, respectively) may on the other hand promote translocation to a lesser extent and over a longer period of time, resulting in increased interaction of NP with the cellular membranes, which generates oxidative stress through membranolytic effects (Figure 6).

We observed that the LDH activity for the SA aerosols compared to controls was only significant for the 5 nm particles (Figure 6). This same aerosol also showed a lower increase in 8-isoprostane concentration than the other two SA aerosols (Figure 6). This could possibly be explained by the higher cytotoxicity response observed in these animals. Indeed, LDH is an enzyme that leaks from damaged cells as a sign of membrane integrity lost [87] and as previously mentioned, is a suitable marker of cell death, particularly by necrosis. It could also be considered as evidence of NP penetration into cells [88]. NP penetration into cells leading to interactions with intracellular components is size-dependent [85,86]. Thus, the lower cytotoxicity observed for the larger NP (10–30 and 50 nm) could possibly be due to their less efficient penetration into cells. Interestingly, our data suggest that membrane damage by lipid peroxidation at the NP – cell membrane interface might not be the primary cause of cytotoxicity. Thus, the size-dependent effect of nano-TiO_2_ observed in our study in the smaller than 100 nm agglomeration state is supported by the literature. Also, these results are in line with Paulhun’s study [11] that reported that the clearance kinetics of NP was more dependent on their initial particle size.

### 50 nm TiO_2_ NP

In addition, for the SA aerosols, there may be a few reasons that explain the lack of cellular and histopathological changes with the 50 nm group compared to the 5 and 10–30 nm groups (Figures 4 and 8). First, considering the NP powder characterization, approximately 20% of the crystal phase of the 50 nm powder was in the rutile form, while it was 3% or less for the two other powders (Table 1). It has already been reported that the rutile form of TiO_2_ NP is less toxic than the anatase crystal phase [38,40,89-92]. Thus, in the less than 100 nm agglomeration state, the presence of the rutile phase in the 50 nm powder may be partly responsible for the lower cellular toxicity observed. At equal mass concentration, as the NP size decreases, the surface area per mass unit increases and leads to high surface to volume ratios, giving smaller NP enhanced surface reactivity [12]. Studies have also shown that the surface adsorption and reactivity of smaller than 10 nm TiO_2_ NP were enhanced relatively to larger NP [19,93]. Hence, the size effect of the initial 5 nm particle size (D_50_ = 48 nm in aerosol), which would be more toxic than the 50 nm particles (D_50_ = 85 nm in aerosol), may also contribute to the cytological effects observed for the SA aerosols. Also, for these three aerosols, the total particle number concentration was elevated (Table 2). However, the 50 nm SA aerosol had the lowest total particle number concentration by a factor of 2.4 and 1.4 compared to the 5 and 10–30 nm aerosols, respectively. Due to their small size, NP mainly contribute to number concentrations in aerosols and, to a much lesser degree, to mass concentration [94,95]. For identical masses, a larger number of NP can occupy the same space, and thus, in theory, increase the interactions with biological material [12,96]. Thus, all of these factors may also contribute to the toxicological results observed for the 50 nm SA aerosol.

### Primary NP size-dependant effect

Overall, these results show that within a less than 100 nm agglomeration state, there may be a primary particle size-dependent effect of nano-TiO_2_. Even though the 10–30 and 50 nm particles induced significantly higher oxidative stress and pro-inflammatory damage than the 5 nm particles, it cannot be directly concluded that these larger TiO_2_ NP are more toxic. Our data, in line with the current literature, show that the smaller 5 nm particles may potentially pose greater health risks by causing more cytotoxicity through necrosis.

It is noteworthy that a limitation to our study can be attributed to the fact that only data 16 hours after a 6-hour exposure were collected. Therefore, conclusions on prolonged inflammation and cytotoxicity cannot be drawn from these data. However, our results indicate that for an acute exposure the 10–30 nm particles induced significant increases in the total cell count and number of macrophages in both the SA and LA aerosols, while the number of neutrophils was significantly increased in the LA aerosol (Table 3, Figure 4), which also showed the highest fold increases in pro-inflammatory cytokine (Figure 5). Moreover, qualitatively comparing the agglomerate structure of the LA aerosols (Figure 2) we noticed that the 10–30 nm particles agglomerated into loose structures with more void open spaces. The possibility of agglomeration and de-agglomeration of NP in physiological environments still remains an open question [3,9,38,45,47]. However, if de-agglomeration was to occur once deposited in the lungs, the loose agglomerate structure is thought to be more easily de-agglomerated [9]. Moreover, when considering the geometry of similar agglomerates size, the loose type structure has a higher surface available to interact with biological materials and could hence increase their toxicity compared to the compact agglomerates. Therefore, the NP agglomerates structure may also play a role in toxicity. Overall, of the three NP sizes, the 10–30 nm TiO_2_ NP seemed to induce the most pronounced pro-inflammatory effects. These results are consistent with Grassian *et al.*[9] inhalation study in mice at 7 mg/m^3^ where it was concluded, solely based on the inflammatory cell response, that the 21 nm nano-TiO_2_ particles (139 nm in aerosol) were slightly, but significantly more toxic than the 5 nm ones (120 nm in aerosol). Interestingly, the highest relative deposition efficiency of NP in the alveolar region occurs at approximately 20 nm [17,18].

## Conclusions

In summary, the results of this study suggest that the initial NP size and the agglomeration state are key determinants of nano-TiO_2_ lung inflammatory reaction, cytotoxic and oxidative stress induced effects. Acute exposure to 20 mg/m^3^ of nano-TiO_2_ in rats inhaled as LA aerosols induced an acute inflammatory response, noted by an increase in the number of neutrophils, while SA aerosols also produced oxidative stress damage and cytotoxicity in BALF. These results indicate that the toxicity modes of nano-TiO_2_ are different when inhaled as two distinct agglomeration states, smaller or larger than 100 nm, and that within a smaller than 100 nm agglomeration state, there may be a primary particle size-dependent effect of nano-TiO_2_. The 5 nm particles caused increased cytotoxic effects while the oxidative damage was milder when compared to 10–30 and 50 nm particles. This suggests that smaller NP can cause cytotoxicity by penetrating more easily into cells and thereby reducing their interaction with the cellular membrane at the NP – cell interface, resulting in decreased oxidative stress effects. In addition, the most pronounced pro-inflammatory effects were induced by the 10–30 nm TiO_2_ NP, which is also the size having the highest relative deposition efficiency in the alveolar region.

The overall observed responses for the LA and SA aerosols as well as the primary particle size effect in the less than 100 nm agglomeration state must be investigated in future studies incorporating multiple doses, time points and nano-TiO_2_ of different crystal phases, as well as systemic and translocation effects to better elucidate the impact of these factors on the NP kinetics and mechanism of toxicity.

## Methods

### General experimental study design

**Figure 10:**
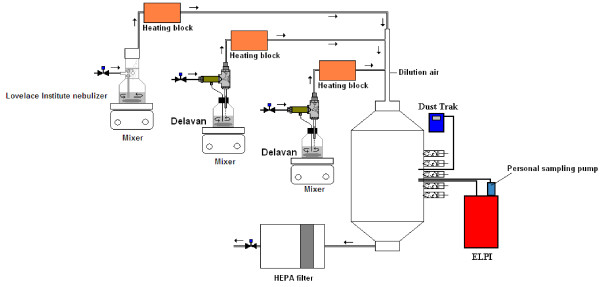
**Experimental set-up for the generation of small agglomerate aerosols.** Experimental set-up using three nebulizers in parallel to produce aerosols essentially composed of small NP agglomerates. ELPI: electrical low pressure impactor; HEPA filter: high-efficiency particulate air filter.

Animal inhalation exposures were performed in a cubic stainless steel 500-L inhalation chamber (Unifab, Kalamazoo, MI) adapted for nose-only. Ports were placed on a single wall of the inhalation chamber so that only the nose of the animal was exposed to the aerosol (see Figure 10). Six groups of rats (n = 6 per group) were exposed to TiO_2_ aerosols for 6 hours; one control group (n = 6) was exposed to clean air for the same duration. One sham group (n = 6) was not exposed and remained housed in the animal facility. No anesthetic treatment was given to the rats prior to the exposures. The aerosols were composed of either 5, 10–30 or 50 nm primary particle size TiO_2_. Each primary particle size of TiO_2_ was generated in aerosols with two distinct size distributions consisting of large (> 100 nm) or small agglomerates (< 100 nm) at mass concentrations of 20 mg/m^3^. Small agglomerate (SA) aerosols were produced by a wet generation method and large agglomerate (LA) aerosols by a dry powder dispersion technique. The animals were sacrificed 16 hours after the end of the exposure period, and bronchoalveolar lavages (BAL) were performed to determine cellular markers of pulmonary toxicity. Lungs were also fixed for histopathology observations.

### TiO_2_ nanoparticles

Anatase TiO_2_ with average particle sizes of 5 nm (Stock# 5420MR) and 10–30 nm (Stock# 5420HT), specific area of 200–220 m^2^/g and near spherical morphologies were purchased from Nanostructured and Amorphous Materials Inc. (Texas, USA). Anatase 50 nm TiO_2_ (MK-TiO_2_-A050) was purchased from MKnano (Ontario, Canada). All the NP were stored in a desiccator placed in a fume hood prior to use. These NP were characterized by TEM (field emission gun (FEG) JEOL JEM-2100F), EDS and XRD (Philips, model: X’Pert, Lelyweg, The Netherlands). These characterization methods were previously described in Noël *et al*. [33].

### Animals

Forty-eight 8-week-old male CDF (F344)/CrlBR (Charles River Breeding Laboratories, St. Constant, Québec, Canada) rats with an average weight on the day of sacrifice of 164 ± 12 g were housed at the animal care facilities of the Université de Montréal. The rats were placed two per cage and had access to water and food *ad libitum*. The animals were exposed to a 12-h/12-h day/night cycle from 6:00 am to 6:00 pm. The animals were acclimatized to the inhalation chamber for a period of six days prior to NP exposure. Weight gain for each animal was recorded daily. The research project was approved by the Université de Montréal’s Ethics Committee on Animal Experiments.

### Generation of TiO_2_ NP aerosols

All aerosols were generated using compressed air that first passed though a Donaldson high-efficiency industrial filter equipped with a coalescing filter (Donaldson DFSP, Series Model-DF 0070 ZU, Ultra-Filter Superplus, Donaldson Company, Inc., Norcross, GA, USA). The average temperature and relative humidity in the inhalation chamber were 23.07 ± 0.72°C and 39.3 ± 16.6% RH. A fan mixed the air in the inhalation chamber. For all exposures, the total air flow entering the chamber was between 3.6 and 4.5 m^3^ per hour.

### Generation of small agglomerate aerosols – Nebulization of TiO_2_ NP

Aerosols composed of small agglomerates were generated using a Lovelace-type nebulizer (In-Tox products, Albuquerque, New Mexico) placed in parallel with two Delavan siphon spray nebulizers (Part number 30609–2 used with an adapter, part number DLN 17147, Delavan Spray Technologies, Goodrich Corporation, Montréal, Canada) (Figure 10). We used three nebulizers in parallel to reach the targeted concentration of 20 mg/m^3^. Indeed, the type and number of nebulizers used constitute important factors contributing to the ultrafine size distribution of the aerosols [33]. The high air flow that passes through the Delavan nebulizer, helps to reduce the size of the droplets and by this means the use of two Delavan allowed the NMAD to be lowered by increasing the number of small NP agglomerates in the aerosol [33]. A 7 g/L (for the 5 nm experiment) or a 5 g/L (for the 10–30 nm and 50 nm experiments) NP suspension in distilled water (Milli-Q reference A+ system, water purification system with total oxidizable carbon indicator, Millipore Corporation) was filtered on Whatman 41 filter paper (Piscataway, NJ, USA) to remove large agglomerates, and sonicated for 10 minutes (Bransonic tabletop ultrasonic cleaner, model 5510, Branson, Danbury, CT, USA). This suspension was poured into the Delavan devices. Since the filtered suspension did not allow the targeted mass concentration to be reached, 2.5 g (for the 5 nm experiment) or 1.5 g (for the 10 – 30 and 50 nm experiments) of the TiO_2_ powder was placed in the Lovelace-type nebulizer prior to the addition of the original suspension (350 ml) to completely fill the device. This new suspension was not sonicated. The suspension was agitated for the entire generation period using magnetic stirring plates placed under each nebulizer. The flow rate used for each nebulizer was 5, 25 and 10–13 L/min for the Lovelace-type nebulizer, the first and second Delavan, respectively. The pressure applied to each nebulizer was between 30 and 35 psi. Dual-element heating tapes (624 watts, 120 VAC, Cole-Parmer, Canada) wrapped around a copper tube were used to dry the aerosol, prior to its dispersion in the inhalation chamber. The flow rate of the dilution air was 25–30 L/min and served to reduce the relative humidity in the chamber created by the water vapor content of the aerosol. No charge neutralization was performed on these aerosols.

### Generation of large agglomerate aerosols –Powder dispersion of TiO_2_ NP

The aerosols composed of large agglomerates were produced using a Fluidized Bed 3400A device (TSI Inc., Shoreview, MN, USA). The exposure concentration for each primary particle size was achieved by adjusting the various feed rates of the Fluidized Bed. The pressure applied to this instrument was between 33 and 38 psi. No charge neutralization was performed.

### TiO_2_ aerosol sampling and characterization

TiO_2_ NP aerosols sampling and characterization methods have been described in detail elsewhere [33]. Briefly, air samples were collected throughout the experiment on cassettes (Sure Seal, SKC Inc.) using 37-mm polyvinyl chloride (PVC) filters at a flow rate of 4 L/min to subsequently determine the average mass concentration by gravimetric analysis. The mass concentrations were followed and adjusted in real time using a Model 8520 Dust Trak Aerosol Monitor (TSI Inc., Shoreview, MN, USA) previously calibrated with TiO_2_ by comparison with the gravimetric method. Air samples were also collected at a flow rate of 1 L/minute on pre-carbon coated Formvar copper grids glued onto 25-mm polycarbonate filters. The glue used was a current cyanoacrylate (Loctite superglue gel, Henkel, Boucherville, Canada). The sampling durations were 2.5 and 5 min for all of the aerosols. Characterization (shape, agglomeration degree and structure) of the aerosols sampled on these grids was performed by TEM (Philips CM200 equipped with a digital camera: Corel Corp. AMTV600 2K×2K, 80 kV). Numbers and particle size distributions were monitored in real time with an electrical low pressure impactor (ELPI) (Dekati Ltd., Tampere, Finland) which was operated at a flow rate of 10 L/minute in the filter stage configuration. The sintered impaction substrates were oiled to prevent or reduce particle bounce. Cumulative size distributions based on number concentration were monitored through 5 thirty-minute samples collected every hour of the experiment. The ELPI was also used to determine the number median aerodynamic diameter NMAD and the geometric standard deviation (GSD). Air samples were all collected in the area of the inhalation chamber corresponding to the breathing zone of the animals.

### Pulmonary deposition modeling

The rats’ airway particle dosimetry was estimated using the Multiple-Path Particle Dosimetry Model (MPPD) (software version 2.11, Applied Research Associates Inc., Albuquerque, NM, USA). The respiratory tract deposition of NP agglomerates was estimated for LA and SA aerosols.

### Bronchoalveolar lavages

The animals were anaesthetized with isoflurane and sacrificed by exsanguination. BAL fluids (BALF) were collected with 0.9% saline. The BAL techniques were previously described elsewhere [97]. Briefly, five 5-ml washes were pooled and placed on ice. The collected BALF were centrifuged at 929 × *g* at a temperature of 4°C for 10 minutes. After centrifugation, the supernatant was removed and frozen at -80°C. These supernatants were aliquoted and used for cytotoxicity and oxidative stress analysis. Multiple freeze–thaw cycle was avoided to prevent loss of enzyme activity. These supernatants were used for cytotoxicity and oxidative stress analysis. The cells were resuspended in 500 μl of saline. 100-μl aliquots were fixed with formalin for cell count (1:1). The lungs were fixed *in situ* with buffered formalin.

### Analysis of the pulmonary response

Cell suspensions were mixed 1:1 with methylene blue to determine the total cell counts using a hemacytometer. The cytospin cell staining method using Hema 3 solutions (Fisher Diagnostics cat. nos. 122-911A, 122-911B and 122-911C) were used to obtain differential cell counts for lymphocytes, neutrophils and macrophages. Slides were observed using a photonic microscope with a magnification of 400-× (Leica DM 1000). Cytotoxicity was evaluated by determining levels of lactate dehydrogenase (LDH) activity (Cytotoxicity Detection Kit for LDH, Roche Applied Science, Laval, QC, Canada). The oxidative stress response was evaluated by measuring 8-isoprostane concentration (8-isoprostane EIA kit, Cayman Chemical, Ann Arbor, MI, USA). Cytokines in BAL were analyzed using Rat Cytokine Array Panel A (R & D Systems, Minneapolis, MN, USA) to determine the relative levels of 29 cytokines (CINC-1, CINC-2α/β, CINC-3, CNTF, Fractalkine (CX3CL1), GM-CSF, sICAM-1 (CD54), IFN-γ, IL-1α, IL-1β, IL-1ra. IL-2, IL-3, IL-4, IL-6, IL-10, IL-13, IL-17, IP-10 (CXCL10), LIX, L-Selectin (CD62L/LECAM-1), MIG (CXCL9), MIP-1α (CCL3), MIP-3α (CCL20), RANTES (CCL5), Thymus Chemokine (CXCL7), TIMP-1, TNF-α, VEGF). To perform this assay, samples from the same exposure group were pooled together. Data from this assay were analyzed by chemiluminescent signals of cytokines/chemokines present in the BALF and were detected on Kodak X OMAT-RA film, as described in Ratthe *et al.*[98] and Gonçalves *et al.*[98]. Results were reported as described in Gonçalves *et al.*[99]. Briefly, cytokines with ≥ 1.2-fold increased compared to the control group were considered to represent a slight inflammation. All assays were performed as specified by the respective manufacturers.

### Estimation of NP endocytosis by macrophages

The cytospin slides were used to visually estimate the extent of NP phagocytosis by counting 200 macrophages for the presence or absence of NP agglomerates inside the cytoplasm. In this way, the percentage of particle-laden macrophages was established. Slides were observed using a photonic microscope (Leica DM 1000).

### Lung histopathology

The lungs were fixed *in situ* with buffered formalin. Subsequently, caudal right lobe lung sections were cut into thin slices (3 to 5 mm thick) using a clean scalpel. Samples were placed inside histology cassettes before processing. Wax infiltration (Sakura Tissue Tek VIP E150) was achieved following dehydration through 3 alcohol baths (70, 85 and 90%) and cleared through 3 toluene baths. For embedding, tissue was oriented inside a mold filled with hot paraffin (Embedding station ESBE EC350). Tissue sectioning (4 μm thick) was done with a microtome (Microtome LEICA RM2255). Sections were then colored using haematoxylin and eosin staining standard protocol. Images were acquired using an Olympus BX51 optical microscope.

### Statistical procedures

BALF cytology, pulmonary cytotoxicity, oxidative stress markers as well as NP endocytosis by macrophages were analyzed using ANOVA and Tukey’s test. Statistical significance was achieved when p < 0.05. Statistical analyses were performed using the Statistical Package for the Social Sciences (SPSS, version 17.0, SPSS Inc.).

## Competing interest

The authors declare that they have no competing interests.

## Authors’ contributions

AN, MC, YC, RT and GT were involved in the conception and design of the study. AN and YC developed the NP aerosols generation and characterization methods. AN and RT were responsible of the inhalation exposures. AN and MC were responsible of the BALF analysis. AN, MC, YC, RT and GT were involved in the analysis and interpretation of data. AN drafted the manuscript. All authors read and approved the final manuscript.
